# Cell Cycle Gene Networks Are Associated with Melanoma Prognosis

**DOI:** 10.1371/journal.pone.0034247

**Published:** 2012-04-20

**Authors:** Li Wang, Daniel G. Hurley, Wendy Watkins, Hiromitsu Araki, Yoshinori Tamada, Anita Muthukaruppan, Louis Ranjard, Eliane Derkac, Seiya Imoto, Satoru Miyano, Edmund J. Crampin, Cristin G. Print

**Affiliations:** 1 Department of Molecular Medicine and Pathology, School of Medical Sciences, Faculty of Medical and Health Sciences, University of Auckland, Auckland, New Zealand; 2 Auckland Bioengineering Institute, University of Auckland, Auckland, New Zealand; 3 Laboratory of DNA Information Analysis, Human Genome Center, Institute of Medical Science, University of Tokyo, Tokyo, Japan; 4 GNI Ltd, Shinjuku-ku, Tokyo, Japan; 5 Bioinformatics Institute, University of Auckland, Auckland, New Zealand; 6 Department of Engineering Science, University of Auckland, Auckland, New Zealand; University of Georgia, United States of America

## Abstract

**Background:**

Our understanding of the molecular pathways that underlie melanoma remains incomplete. Although several published microarray studies of clinical melanomas have provided valuable information, we found only limited concordance between these studies. Therefore, we took an *in vitro* functional genomics approach to understand melanoma molecular pathways.

**Methodology/Principal Findings:**

Affymetrix microarray data were generated from A375 melanoma cells treated *in vitro* with siRNAs against 45 transcription factors and signaling molecules. Analysis of this data using unsupervised hierarchical clustering and Bayesian gene networks identified proliferation-association RNA clusters, which were co-ordinately expressed across the A375 cells and also across melanomas from patients. The abundance in metastatic melanomas of these cellular proliferation clusters and their putative upstream regulators was significantly associated with patient prognosis. An 8-gene classifier derived from gene network hub genes correctly classified the prognosis of 23/26 metastatic melanoma patients in a cross-validation study. Unlike the RNA clusters associated with cellular proliferation described above, co-ordinately expressed RNA clusters associated with immune response were clearly identified across melanoma tumours from patients but not across the siRNA-treated A375 cells, in which immune responses are not active. Three uncharacterised genes, which the gene networks predicted to be upstream of apoptosis- or cellular proliferation-associated RNAs, were found to significantly alter apoptosis and cell number when over-expressed *in vitro*.

**Conclusions/Significance:**

This analysis identified co-expression of RNAs that encode functionally-related proteins, in particular, proliferation-associated RNA clusters that are linked to melanoma patient prognosis. Our analysis suggests that A375 cells *in vitro* may be valid models in which to study the gene expression modules that underlie some melanoma biological processes (e.g., proliferation) but not others (e.g., immune response). The gene expression modules identified here, and the RNAs predicted by Bayesian network inference to be upstream of these modules, are potential prognostic biomarkers and drug targets.

## Introduction

### Clinical aspects of melanoma

Malignant melanoma is a devastating form of cancer with a particularly high incidence in New Zealand (NZ) and Australia [1]. Although early-stage melanoma is curable, advanced melanoma is very difficult to treat and is comparatively resistant to chemotherapy. Very few agents (e.g. interferon-alpha2b) are useful as adjuvant chemotherapy after primary tumours have been excised. For disseminated melanoma there are currently only a small number of chemotherapeutic agents in general use (e.g. temozolomide and dacarbazine), which are not effective in all patients [2]. Emerging approaches such as BRAF inhibition (PLX4032, [3]) and immune-based therapies ([4]–[8]) hold great promise, but are unlikely to be effective for all melanoma patients. We urgently need to improve our understanding of the complex and variable molecular pathogenesis of melanoma, and based on this understanding, develop biomarkers to allow better matching of patients to therapeutic approaches. This study attempts to address this challenge.

### Melanoma molecular pathways

The molecular pathways that underlie melanoma are complex. The roles of twenty-five molecules strongly associated with malignant melanoma are summarised as briefly as possible below, so that when functional genomic approaches based on mRNA data are used later in this study, we can assess whether these molecules and the molecular pathways they constitute are identified.

Inherited mutations cause a genetic predisposition to melanoma, including mutations in cell cycle genes such as *CDKN2A* [9], *CDK4* [10], *RB1* [11] and *MDM2* [12], as well as melanocyte differentiation and activation genes such as *MC1R*, *TYR*, *TYRP1* and *ASIP* [13]. Somatic mutations in other genes are thought to play a role in disease progression. For example, phosphatidylinositol 3-kinases (PI3K) and their downstream targets of the protein kinase B (Akt) family are constitutively activated in many melanomas [14]. The gene encoding the phosphatase PTEN is also commonly mutated in melanoma [15], which reduces PTEN's ability to dephosphorylate phosphoinositides and to inhibit PI3K-Akt signalling pathways, and therefore increases proliferation and decreases apoptosis [16].

Other molecules commonly involved in melanoma progression include NRAS[17] and BRAF [18], [19], which appear to occur in mutually exclusive sets of tumours [20] and lead to constitutively active MEK–ERK signalling. This causes up-regulation of p38/Jun N-terminal kinase (JNK) activity and activation of c-Jun, which promotes the transcription of Jun targets including *MMP2*, *RACK1* and *CCND1* [21]. Constitutively active MEK–ERK signalling also causes phosphorylation and activation of the transcription factor MITF [22], which promotes the expression of its target genes including *BCL2*, *CDKN1A*, *TYR*, *TBX2* and *CDK2* [23], [24]. *MITF* expression is also promoted by transcription factors such as Pax3 and Sox10 [25] and inhibited by the transcription factor BRN2 [26]. The overall expression of *MITF* in melanoma is associated with clinical outcome [27], however, melanomas are heterogeneous, appearing to contain individual cells with different phenotypic and gene expression patterns [28]. When *MITF* expression in melanomas is examined on a cell by cell basis, the slow-growing stem-cell-like melanoma-initiating cell population appears to have low *MITF* expression, and in accord with this, inhibition of MITF in B16 mouse melanomas reduces proliferation and up-regulates the stem cell marker Oct4 [29]. It appears that the BRAF and MITF signalling pathways described above synergise to give melanoma cells their neoplastic, and later their invasive and metastatic, phenotypes. For example, p16^INK4^ inactivation and *BRAF* mutation can accompany *MITF* amplification in melanoma cell lines, and ectopic *MITF* expression appears to work in synergy with *BRAF* mutation to transform primary human melanocytes [30].

### Inferring molecular pathway activity from gene expression data

Melanoma research was one of the earliest fields in which expression profiling was applied to tumour classification [31]. RNAs over-expressed in melanoma have been used to predict melanoma invasiveness, metastasis, prognosis and immunotherapy response, and are thought to represent transcriptional signatures of some of the melanoma molecular pathways described above [32]–[36]. The abundance of RNAs encoding proteins that are targets of the same transcription factors [37] or that function within the same molecular pathways [38] are sometimes correlated in an evolutionarily conserved and tissue-specific manner [39], [40]. Therefore the activity of signalling pathways may potentially be inferred from the abundance and correlation of those RNAs known to be transcribed when the pathways are active [41], [42]. This principle has been used to identify molecular pathways associated with the transformation of melanocytes into melanomas [43], and contributes to *in silico* models of gene-to-gene relationships known as gene networks [44]. In a gene network, a connection between two RNAs (sometimes referred to as an “edge”) implies either co-expression of the two RNAs or the regulation of the abundance of one RNA by the abundance of the other, either directly or via intervening signalling molecules and transcription factors. In gene networks RNAs are usually referred to as “nodes”, connections between them referred to as “edges” and groups of RNAs that are highly correlated with one other are referred to as “clusters”. There are several types of gene networks that model RNA-to-RNA relationships using different assumptions, ranging from simple non-directional correlation-based methods [39], sometimes referred to as relevance networks, to complex Bayesian gene networks, which can model directional and synergistic relationships between molecules [45], [46]. Until recently, due to computational limitations, most directional gene network methods could only model interactions between a few hundred genes at a time. However, in 2010, a method to identify whole-genome-scale Bayesian gene networks using massively parallel supercomputers was developed [47], which is used in this study.

### Combination of cell line and tumour gene expression data in this study to understand melanoma pathways

In this study we find that the association of tumour clinical features with either individual RNAs or inferred molecular pathway activity is not consistent across published melanoma microarray datasets. Given this lack of consistency, and the consequent difficulty of using data from the diverse melanomas of patients to understand melanoma molecular pathways, we instead take an *in vitro* functional genomic approach. We generate microarray data from the melanoma cell line A375 exposed to a set of targeted siRNA disruptions, and used these data to identify co-expressed clusters of genes that are strongly conserved between siRNA-treated A375 cells and melanomas from patients. Several of these individual clusters encode proteins with shared cellular functions; we show that those clusters related predominantly to cellular proliferation are significantly associated with the prognosis of metastatic melanoma patients.

## Results

### Published melanoma studies fail to identify consistent gene or molecular pathway signatures

**Figure 1:**
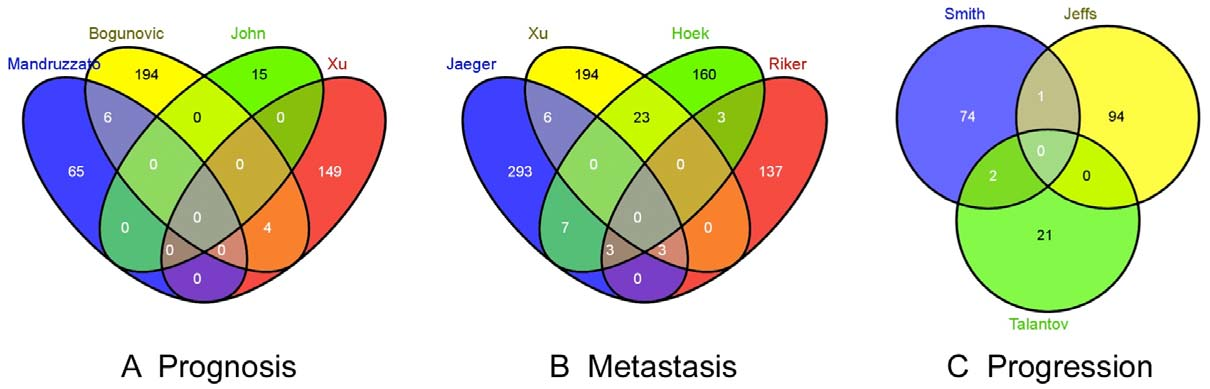
Intersection between gene signatures identified by microarray studies of melanoma tumour prognosis, metastasis and invasion. (A). The intersection between RNAs identified in four studies of *prognosis* ([78];[79];[56];[57]). (B). The intersection between RNAs identified in four studies of *metastasis* ([56];[28];[54];[55]). (C) The intersection between RNAs identified in three studies of *invasion* ([80];[81]; [82]).

In several individual published microarray studies of melanomas from patients, sets of genes appear to be differentially expressed in association with three aspects of tumour biology: *progression*, *metastasis* and *prognosis*. We wished to assess whether the genes associated with these clinical features were consistent across the multiple published studies. Therefore, the raw data from several well-designed microarray studies that addressed *progression*, *metastasis* and *prognosis* were retrieved (Table 1). Quality control assessment indicated that all data was of acceptable quality and re-analysis of each dataset from Table 1 identified sets of differentially expressed RNAs similar to those previously published, although the different studies appeared to vary widely in their statistical power. However, a statistical meta-analysis using the R ‘metaMA’ package with false discovery rate controlled to ≤5% [48] was unable to identify any sets of RNAs consistently associated with *progression*, *metastasis* or *prognosis*. Venn diagrams are shown in Figure 1 to illustrate the relative lack of consistency between RNAs differentially expressed in the various studies.

**Table 1:** Gene signatures from multiple microarray studies of melanomas from patients.

Author	Melanoma samples	Prognostic signatures	Progression signatures	Metastatic signatures	Reference
Winnepenninckx et al.	primary melanoma	+	+		[58]
Mandruzzato et al.	metastatic melanoma	+			[78]
Riker et al. (GSE7553)	primary vs. mixed metastatic			+	[55]
Jaeger et al.	primary vs. cutaneous metastatic			+	[54]
Haqq et al.	primary vs. mixed metastatic		+	+	[83]
John et al.	lymph node metastases	+			[79]
Xu et al. (GSE8401)	melanoma tumours	+		+	[56]
Pfaff-Smith et al.	primary vs. mixed metastatic		+	+	[80]
Jeffs et al.	melanoma cell lines		+		[82]
Hoek et al.	melanoma primary cultures			+	[28]
Talantov et al.	normal skin vs nevus vs primary melanoma		+		[81]
Bogunovic et al.	metastatic melanoma	+			[57]

The table summarises melanoma prognostic, progression and metastatic gene signatures that have been generated from a set of high-quality published microarray studies.

+ indicates that the array study generated the corresponding type of signature.

We then used pathway level analysis to analyse the differential expression of functionally-linked gene sets associated with melanoma *progression*, *metastasis* and *prognosis*. The *Gene Annotation Tool to Help Explain Relationships* (GATHER) [49] and *Principal Coordinates and Hotelling's T2* (PCOT2) [42] applications failed to find any TRANSFACPro [50], Gene Ontology (GO) [51], or Kyoto Encyclopaedia of Genes and Genome (KEGG) [52] gene sets that were consistently differentially expressed in more than one study of melanoma *prognosis* or *progression*. GATHER analysis only identified two gene sets consistently differentially expressed in the studies of metastasis (G-protein coupled receptor signalling, GO.0007186 and epidermis development, GO.0008544; Bayes Factor ≥5, p≤0.05). In summary, our analysis of published melanoma microarray studies addressing tumour *progression*, *metastasis* and *prognosis* identified little concordance between the different studies at the levels of individual RNAs or gene sets.

### Generation of a microarray dataset using siRNA knockdowns in cultured A375 melanoma cells

**Figure 2:**
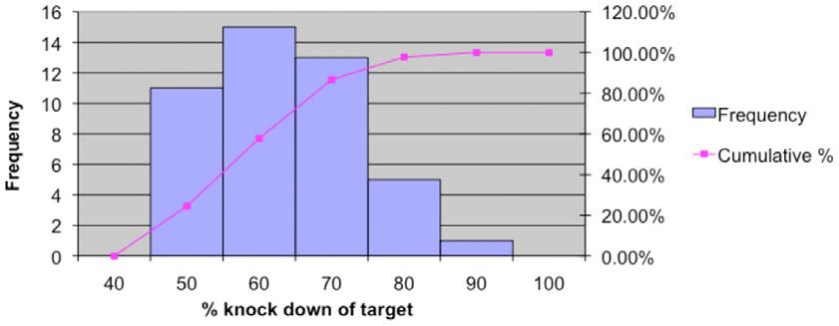
Frequency distribution of % ‘knock-down’ of target mRNA abundance. X-axis represents the percentage knock down in mRNA target abundance that was induced by the specific siRNA, as reported by microarrays (calculated based on mRNA abundance in the siRNA-targeted array/median mRNA abundance in all other arrays). The left y-axis represents the frequency of knockdown for each of the x-axis bins (blue bars) and the right y-axis represents the cumulative frequency (pink line).

Given the lack of consistent RNA signatures for melanoma progression, metastasis and prognosis from microarray studies of melanomas from patients, and the consequent difficulty of using data from the diverse melanomas of patients to understand melanoma molecular pathways, we took an *in vitro* functional genomics approach. This involved multiple siRNA knockdown experiments in the A375 melanoma cell line, in which the abundance of specific target mRNAs were reduced in separate cultures of melanoma cells before Affymetrix U133plus2 microarray analysis. The principle of this study was that each siRNA experiment would alter the activity of a subset of signalling pathways and consequently the abundance of mRNAs downstream of those pathways, allowing clustering and gene network analysis to identify the strongest statistical relationships between any of the 54,000 probe sets, across the siRNA-treated cells. We selected 45 siRNAs (Table 2) that targeted molecules known to be important in melanoma cell biology and were able to produce ≥2 fold reduction in the abundance of their target mRNAs. Additional selection criteria were: (i) that the target molecules were recorded in the Ingenuity Pathways Analysis (IPA) systems biology database (http://www.ingenuity.com/) to influence the expression of ≥50 downstream mRNAs, and (ii) they were relatively abundant in A375 cells (on average ≥50^th^ percentile of abundance in the microarray data). RNA from A375 cultures transfected with these 45 siRNAs, along with control inactive fluorescently-labelled siRNAs, were analysed using Affymetrix microarrays. The distribution of target ‘knock-down’ efficacy is shown in Figure 2.

**Table 2:** siRNA targets.

OGS	degree of target knockdown (relative to the median array)	% reduction in expression	Batch
ABL1	−2.01	50%	KD2
AKT1	−2.79	64%	KD2
CCNA2	−2.87	65%	KD2
CCNB1	−2.84	65%	KD2
CCNB2	−3.96	75%	KD3
CCND3	−2.91	66%	KD2
CDC16	−2.98	66%	KD2
CDC2	−2.72	63%	KD1
CDC25B	−2.08	52%	KD1
CDC37	−2.17	54%	KD2
CDK2	−2.6	62%	KD2
CDK4	−3.42	71%	KD2
CDK7	−2.53	60%	KD1
CDKN2C	−2.37	58%	KD2
CEBPD	−2.02	50%	KD2
CEBPZ	−2.96	66%	KD2
CHEK1	−2.8	64%	KD2
CTNNB1	−2.19	54%	KD1
ETS1	−1.98	49%	KD3
FOXM1	−2.69	63%	KD2
FOXO3A	−2.72	63%	KD1
GABARAP	−3.6	72%	KD2
HDAC2	−2.84	65%	KD2
HDAC3	−2.77	64%	KD2
HSF2	−4.62	78%	KD1
MAP2K1	−3.44	71%	KD2
MAPK1	−2.05	51%	KD2
MCM2	−5.96	83%	KD3
MITF	−14.51	93%	KD3
NCOR2	−2.32	57%	KD2
NMI	−4.77	79%	KD2
PCNA	−2.7	63%	KD1
PIAS1	−3.04	67%	KD3
PIK3CB	−2.15	53%	KD2
RB1	−4.34	77%	KD1
RBL2	−2.53	60%	KD2
RELA	−2	50%	KD2
SKP2	−3.4	71%	KD2
SP1	−1.99	50%	KD3
SP100	−3.06	67%	KD2
STAT1	−3.29	70%	KD2
STAT3	−5.75	83%	KD3
STAT6	−2.21	55%	KD2
TCEA1	−3.04	67%	KD1
TP53	−2.74	64%	KD1

‘OGS’ designates the official gene symbol of the target mRNA, ‘Degree of knockdown’ is the fold reduction in expression of the target RNA after siRNA incubation relative to median expression of the target RNA in all microarrays, ‘% reduction in expression’ is the % that the target RNA expression is reduced relative to median expression of the target RNA in all other microarrays, and ‘Batch’ is the experimental batch in which the siRNA was used.

### Identification of biologically-relevant clusters in the A375 microarray dataset using unsupervised methods that make no prior assumptions about cluster membership

To explore whether this A375 dataset contained biologically sensible information, we first attempted to identify biologically relevant clusters of RNAs that were correlated across the A375 siRNA microarray dataset. As discussed in the introduction, we would expect mRNA targets of transcription factors [37] or mRNAs encoding proteins of common function [53] to be more highly correlated than expected due to chance. Hierarchical clustering was performed in R using Ward's method and the dendrogram cut to identify 200 clusters of probe sets (see Methods). Each cluster was then analysed using the GATHER web tool accessed through an R script to identify any GO or TRANSFAC gene sets for which clusters were significantly enriched. 66 clusters with at least one enriched gene set were identified; eight of these were significantly enriched for the targets of specific transcription factors (Table 3) and five were significantly enriched for cell cycle-associated GO gene sets (GO paths GO:0007049 {3 clusters} and GO:0008283 {2 clusters}). GO paths for which other clusters were enriched included: DNA recombination (GO:0006310), transcription (GO:0006350), protein folding (GO:0006457), intracellular apoptosis induction (GO:0008629) and regulation of phosphorylation (GO:0042325). Similar results were obtained when we identified stably observed clusters using bootstrap resampling through the ‘pvclust’ R package; we found 134 clusters with ‘approximately unbiased’ *p*-values ≥0.95, 51 of which had at least one enriched GO path; of these seven had functional enrichment for cellular proliferation.

**Table 3:** Enrichment of transcription factor targets in A375 clusters.

Cluster	TRANSFAC Annotation	Number of RNAs in cluster with annotation	ln Bayes factor	probability of obtaining ≥ this Bayes factor by chance
9	V$CDC5_01: cell division control protein 5	65	5.37	0.01
9	V$E2F1_Q6: E2F-1	61	6.51	0.01
79	V$SOX5_01: Sox-5	32	7.62	0
79	V$SOX9_B1: SOX (SRY-related HMG box)	35	6.46	0.01
101	V$KROX_Q6	61	10.56	0
101	V$MAZ_Q6	71	5.84	0
101	V$MAZR_01: MAZ related factor	50	4.27	0.02
113	V$CMYB_01: c-Myb	9	6	0.04
246	V$YY1_02: Yin and Yang 1	13	5.31	0.05
266	V$FOXO4_01: fork head box O4	24	5.11	0.02
282	V$WHN_B: winged-helix factor nude	10	6.49	0.03
283	V$OCT1_02: octamer factor 1	13	7.29	0.01
358	V$NRF1_Q6	5	6.27	0.04

The first column is the cluster identifier. The second column is the enriched TRANSFAC Pro v8.2 transcription factor motif in the promoter of the genes in the cluster. The third column is the numbers of RNAs with the transcription factor target annotation in the cluster. The fourth and fifth columns are from the GATHER web tool – indicating the Bayes factor and the permutation *p*-value for the Bayes factor (indicating how often ≥ this Bayes Factor may be expected due to chance), respectively.

**Figure 3:**
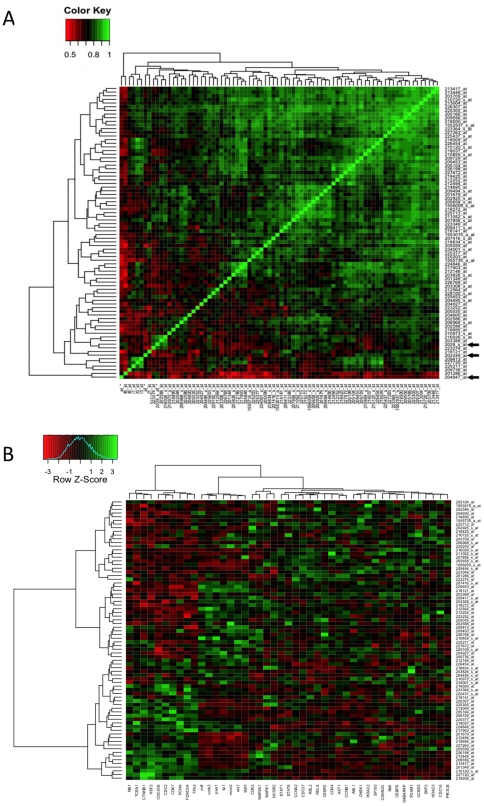
Heatmaps illustrating relationships across data from A375 cells treated with siRNAs *in vitro* between members of a cluster of mRNAs found to be enriched for an E2F1 promoter motif. (A) Heatmap illustrating Spearman's correlations within the cluster. The colour key at the top left maps Spearman's correlation coefficients between probe sets to colour, note that the range of ρ is +0.4 to +1. Probe sets encoding E2F-family proteins are indicated by arrows. (B) Heatmap illustrating expression values of probe sets in this cluster (rows) in the A375 siRNA knockdown arrays (columns), the colour key at the top left maps Z-transformed expression values to heatmap colours.

In one particular cluster, 61 of 67 RNAs were targets of the cell cycle-associated transcription factor E2F1. This is shown by plotting heatmaps of Spearman's correlation coefficient (ρ) and gene expression (Figure 3). Many of the RNAs represented in this cluster were also correlated (or negatively correlated) with the RNAs encoding the E2F family members themselves (which were also part of the cluster, and are arrowed in Figure 3A). This cluster is also significantly enriched for genes that encode members of the cell cycle-associated GO gene set GO:0007049.

**Figure 4:**
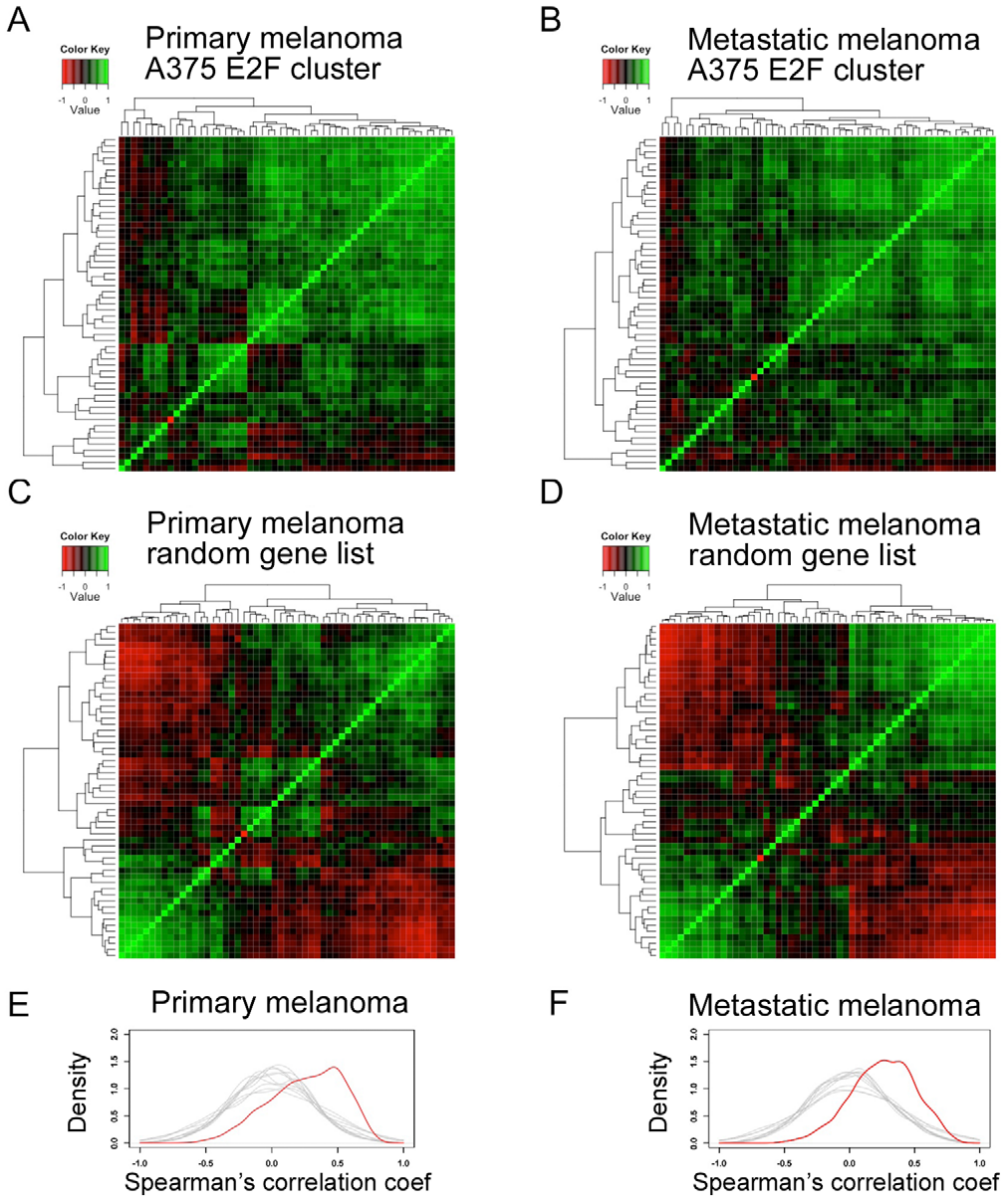
RNAs that are correlated in an E2F1-associated A375 cell-derived cluster are also correlated in datasets of primary and metastatic melanomas. A and B show heatmaps of Spearman's correlation coefficients between the E2F1 cluster probe sets shown in Figure 3, across primary and metastatic melanoma data. C and D show heatmaps of Spearman's correlation coefficients between members of a random list of probe sets the same size as the E2F cluster used in panels A and B, across primary and metastatic melanoma datasets. E and F show in red kernel density plots of the correlations shown in A and B, respectively. In grey they show Spearman's correlations across primary and metastatic melanoma datasets, respectively, between 10 random list of probe sets the same size as the E2F cluster. The colour key at the top left of each heatmap maps Spearman's correlation coefficient between probe sets to colour. The deepest red colour represents the Spearman's correlation coefficient of −1 and the deepest green colour Spearman's correlation coefficient of 1. Note that, in order to illustrate the broad range of correlations observed, the scale used here is different from that used in Figure 3.

To evaluate the potential clinical relevance of these clusters, we assessed whether the RNAs we identified as co-expressed across the A375 siRNA microarray data were also co-expressed across microarray data from primary and metastatic melanomas from patients. A composite Affymetrix U133A microarray dataset from primary melanomas using three published studies [54]–[56], and a separate composite Affymetrix U133A dataset from metastatic melanomas using four published studies [54]–[57] were assembled and normalised from raw data by the RMA method. Ward's method hierarchical clustering using the Agnes function in R with either: (i) all probe sets or (ii) only those probe sets with median signals ≥1.5x the 3′ BioB probe set (i.e. well above the level of noise in the microarrays) suggested that the A375 cell data lay approximately equidistant between the primary and metastatic melanoma data sets, which were more similar to one another than they were to the A375 cell data (data not shown). Using the probe sets from the A375 cell cluster that is shown in Figure 3, we calculated Spearman's ρ across both the primary and the metastatic tumour microarray data (Figure 4 A and B, respectively). As a control, Spearman's ρ was also calculated across the tumour data for equally sized but randomly chosen RNA sets (Figure 4C–D). For these randomly chosen RNA sets relatively few RNAs were seen to correlate highly with one another (Figure 4E–F). Similar results were found for clusters enriched for SOX9, FOXO4 and MAZ targets. For these gene sets, in primary melanoma data 43%, 32% and 39% of possible probe set pairs, respectively, had Spearman's ρ≥0.6. In metastatic melanoma data 56%, 61% and 31% of possible probe set pairs, respectively, had Spearman's ρ≥0.6.

In summary, unsupervised clustering of microarray data from siRNA-treated A375 cells identified RNA clusters that encoded proteins with common functional annotations, as well as RNA clusters that shared common transcription factor binding motifs in their gene promoters. We showed that members of four of these A375 cell-derived clusters were also correlated in both primary and metastatic melanomas, suggesting that the A375 microarray dataset did indeed contain ‘biologically sensible’ and clinically valid information.

### Systematic analysis in the A375 data of gene sets associated with specific transcription factors or biological functions

We then screened pre-defined gene sets that shared common transcription factor promoter motifs (based on the TRANSFAC Pro database) or functional annotations (based on the GO database) for high correlations in any of: (i) the siRNA-treated A375 dataset, (ii) primary melanomas, and (iii) metastatic melanomas. Gene sets with high correlations between their constituent RNAs in both the A375 and clinical melanoma data may represent cases where the siRNA-treated A375 data can provide a valid model for gene regulatory processes that occur in the melanomas of patients. Conversely, gene sets with high correlations between their constituent RNAs in clinical melanoma microarray data but not in the A375 data may represent cases where clinically important RNA clusters are not adequately modelled by our siRNA-treated A375 cells.

**Figure 5:**
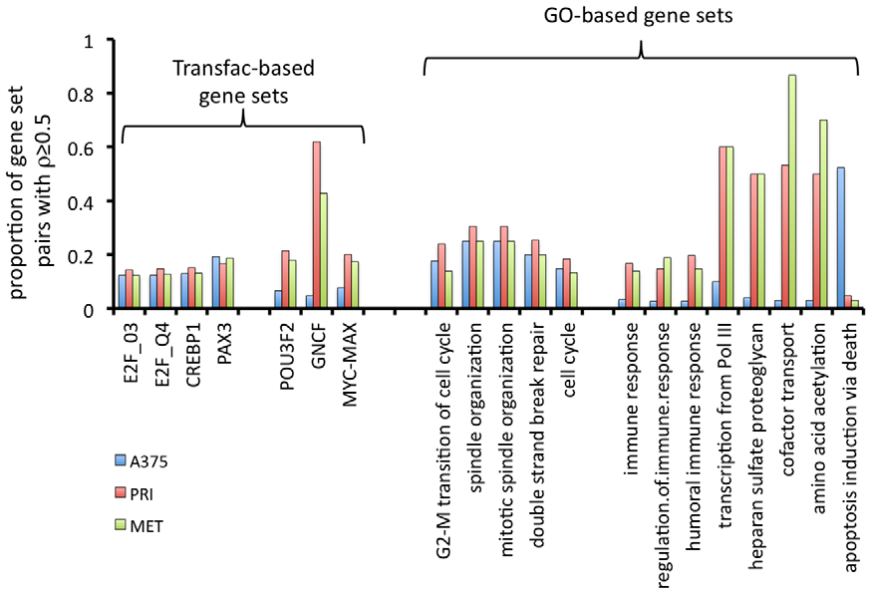
Correlations within functionally-related gene sets in A375 cells and melanomas. All possible gene-gene correlations within gene sets defined by TRANSFAC and GO were calculated. The proportion of the gene pairs within each gene set that had Spearman's |ρ|≥0.5 was calculated separately for A375 siRNA microarray data (blue bars), a composite Affymetrix dataset from three primary melanoma studies (red bars), and a composite Affymetrix dataset from four metastatic melanoma studies (green bars). The x-axis represents TRANSFAC or GO gene sets. The y-axis represents the proportion of gene pairs from each gene set that had Spearman's |ρ|≥0.5 for the A375 data, primary tumour data, and metastatic tumour data, respectively.

All possible gene sets with ≥5 members were retrieved from: (i) the TRANSFAC Pro v 8.2 database (356 gene sets, each containing genes with a common transcription factor binding motif in their promoters) and (ii) the GO v 1.81 database (1,229 gene sets, each encoding proteins with common function). For each TRANSFAC and GO gene set, using each of the three microarray datasets, we used an R script to calculate the fraction of gene pairs that correlated so that the absolute value of Spearman's correlation coefficients (|ρ|) was ≥0.5. We found that several gene sets had a similar, albeit relatively low, frequency of correlated gene pairs in all three of the A375, primary and metastatic melanoma datasets – e.g. E2F1, PAX3, CREBP, cell cycle and DNA repair gene sets (Figure 5). We also found gene sets with members that were more frequently correlated across tumours than across the A375 microarray data. These included immune response, heparin sulphate proteoglycan synthesis, amino acid acetylation, MYC/MAX, POU3F2 and GCNF gene sets (Figure 5). Interestingly, a GO gene set associated with death receptor-induced apoptosis was more frequently correlated in the A375 microarray than in primary or metastatic tumours (Figure 5).

In summary, we identified several gene sets, including gene sets associated with the cell cycle, that had similar frequencies of correlation in both siRNA-treated A375 cells and in melanomas from patients. These may represent active transcriptional pathways regulating biological processes that occur in melanomas and appear to be effectively modelled in siRNA-treated A375 cells *in vitro*. However, transcriptional pathways underlying some other processes such as immune response appear to be identified across the melanoma tumours (in which complex tumour cell-leukocyte interactions occur) but are not apparent in the microarray data from A375 cells cultured in the laboratory.

### Bayesian gene network analysis

In order to predict more complex RNA-to-RNA relationships in the siRNA-treated A375 cells, including upstream regulators of the co-expressed clusters described above, and the putative direction of RNA-to-RNA relationships, we analysed the A375 cell microarray data using a whole-genome Bayesian gene network inference method [47], which identified 1,645,882 edges (can be downloaded, with a brief explanatory file, from http://www.bioinformatics.auckland.ac.nz/doc/project_data/Supplementary_FIle_1.txt).

As described in the introduction, gene network nodes with large numbers of downstream “children” are putative master-regulators of biological processes, and are often known as “hubs”. 11 of the 25 molecules described in the introduction as important for melanoma pathogenesis were identified as hubs in the Bayesian gene network: *SOX10*, *CCND1*, *RB1*, and *BCL2* all had over 50 children, while *PTEN*, *TYR*, *CDKN2A*, *BRAF*, *PAX3*, *AKT1* and *MITF* had between 30 and 50 children. In line with the clustering analysis described above, several members of the E2F transcription factor family were hubs in the gene network, with *E2F4* (38707_r_at), *E2F7* (241725_at), and *E2F1* (2028_s_at) having 181, 103 and 43 network children, respectively. Reassuringly, 226 of the 327 combined children of these three E2F transcription factors have E2F binding sites in their promoters, a significantly greater proportion than would be expected due to chance (empirical p≤0.05). As well as identifying hubs, Bayesian gene networks also identify clusters of co-expressed RNAs, which are downstream of the same hub. Identifying these clusters may be seen as a more conservative use of this network method than identifying directional edges, and is the primary use made of Bayesian gene networks in this paper. Reassuringly, every one of the 200 clusters identified by the hierarchical clustering method above had at least 70% of their members included among clusters identified by the gene network method.

### Are Bayesian gene network hubs and clusters associated with melanoma patient survival?

We wished to determine whether the Bayesian gene network hubs and clusters identified from the A375 microarray data were associated with prognosis. Therefore, we used the ‘Survival’ package in R to generate Cox proportional Hazards models to estimate the association between the abundance of RNAs in tumours and the survival of melanoma patients. Two survival models were generated: (i) based on gene expression in metastatic melanomas using an Affymetrix microarray dataset [57] and (ii) based on gene expression in primary melanomas using an Agilent microarray dataset [58], which was mapped to Affymetrix probe IDs using Entrez gene ID annotations. We then used this melanoma microarray survival information to assess whether gene network hubs and clusters were significantly associated with patient survival.

**Figure 6:**
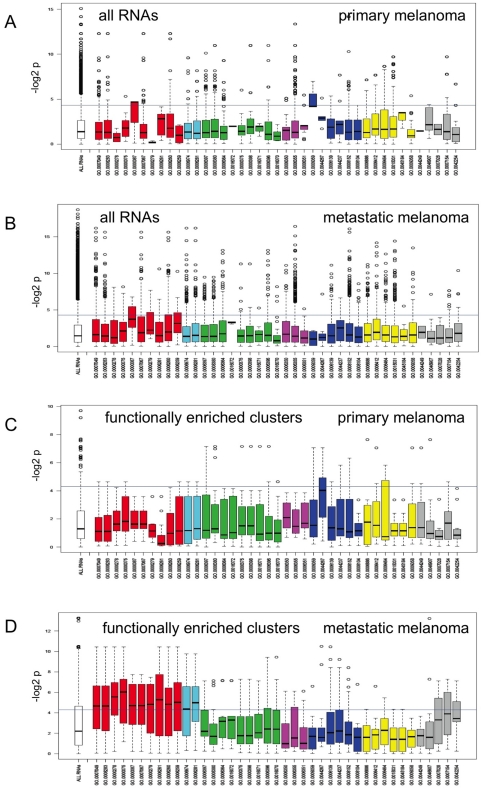
Association between melanoma patient survival and the abundance of RNAs identified as gene network hubs that have their gene network children enriched for specific GO annotation categories. The x-axes represent GO annotations. GO annotations with related functions are coloured and grouped together: red (cell cycle related); sky blue (DNA repair related); green (RNA related); purple (transcription related); dark blue (metabolism related); yellow (protein related) and grey (miscellaneous). The left-most box (white) in each panel represents all RNAs on the microarray. The y-axes represents minus log_base2_ p value, based on a Cox proportional hazards model, to indicate the strength of association between RNA abundance and patient survival. The horizontal line (y = 4.32) represents a threshold for a significant association with survival (equivalent to p = 0.05, above this line is a significant association). Within each box the dark horizontal line represents the median, the coloured area of the box the interquartile range. Panels A and B include all RNAs on the microarrays that have the specified GO annotations. Panels C and D include the Bayesian network hub RNAs, for which the function of downstream gene network children is significantly enriched for the specified GO annotations. Panels A and C represent primary tumours [58] while panels B and D represent metastatic tumours [57].

Firstly, to establish a baseline, we considered whether the abundance of RNAs that encoded proteins with particular classes of functional annotation were significantly associated with patient survival. We hypothesised that RNAs encoding the types of proteins that perform important oncogenic functions (e.g. invasion, DNA replication, or immune response) may be more strongly associated with the survival of patients than the abundance of RNAs that encode proteins that do not play known roles in cancer. For both primary tumours (the Winnepenninckx *et al*., 2006 dataset [58], Figure 6A) and metastatic tumours (the Bogunovic *et al*., 2009 dataset [57], Figure 6B), no one functional category was clearly more or less associated with patient survival than all RNAs taken together. This analysis was repeated for all Bayesian gene network hubs with ≥50 downstream children but again it did not identify any particular functional category with strong patient survival associations (data not shown).

We then repeated this analysis focussing on hubs *with children that encoded proteins of common function*. We used the GATHER web tool to identify hubs with children significantly (Bayes Factor ≥5 and p≤0.05) enriched for GO paths. We found that 204 hubs had children significantly enriched for one of 60 GO paths, which covered a broad range of functions including transcription, metabolism, signal transduction, stress response, DNA repair, and cellular proliferation. Box plots were used to visualise the strength of association between the expression of these hub mRNAs in primary (Figure 6C) and metastatic (Figure 6D) melanomas and patient survival. Functional enrichment of children had little influence on the strength of association between hub mRNA abundance and patient survival in the Winnepenninckx *et al*. primary tumour dataset (Figure 6C), however, it appeared to have a strong influence on the strength of association between hub abundance and patient survival in the Bogunovic *et al*. metastatic tumour dataset (Figure 6D). For example, 64% of the hubs that had their children enriched for cell cycle regulation functions had statistically significant associations with patient survival. Interestingly, in all cases where hubs had children enriched for cell cycle functions, the hub itself also encoded a protein with cell cycle function. Conceivably, by providing a summary of the abundance of their cell cycle-related co-expressed children, these hubs may in effect be quantifying the activity of cell cycle pathways in metastatic melanoma tumours.

**Figure 7:**
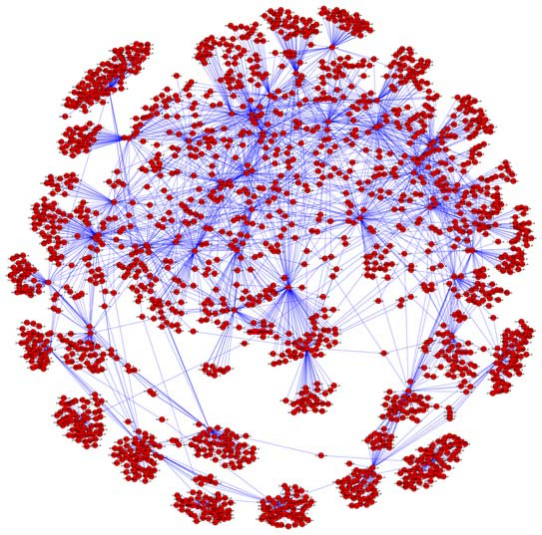
The edges immediately downstream of gene network hubs that were enriched for cell cycle-associated children. Each red dot represents a probe set of either a gene network hub that has significant cell cycle enrichment of children, or one of the children. Blue lines represent gene network edges.

The hubs with children enriched for cell cycle functions included: *MCM5* (initiation of DNA replication – this cluster has 73% intersection with the *E2F1*-associated cluster identified by simple clustering analysis and shown in Figure 3), *TYMS*, *DTL*, *CENPU*, *PRIM1*, *MELK1* and *PBK* (PDZ binding kinase, a serine/threonine kinase). All hubs with children enriched for various GO paths related to cell cycle, mitosis or proliferation with Bayes Factor ≥5 and p≤0.05 are shown in Table S1. Displaying the edges between the cell cycle-associated hubs and their children as a directed graph using the Cytoscape application showed that most of the cell cycle-associated clusters were relatively independent of one another, although some were extensively interlinked (Figure 7).

Several of these A375 gene network hubs and downstream cell cycle-associated clusters are potentially clinically relevant, since hub RNA-to-child RNA correlations were found in both the A375 and metastatic tumour (Bogunovic [57]) data set. For example, 16 of the A375 data-derived network hubs with cell cycle-enriched children also had Spearman's correlations of |ρ|≥0.4 with ≥10 of their children across the tumours in the Bogunovic clinical melanoma data set (Table S2). As an example, the Spearman's correlations in the A375 and Bogunovic datasets between the hub *DTL* and its gene network children are listed in Table S3.

### Gene network hubs used to classify melanoma patient outcome

Given that a subset of the A375 gene network hubs appeared to be clinically relevant molecules, we used those 181 gene network hubs with ≥50 children and with an association with metastatic melanoma patient survival of p≤0.005 to construct a classifier for patient survival. This was done using metastatic melanoma microarray data [57], after separating tumours into two classes: (i) tumours from patients who died before 2 yrs (n = 10), and (ii) tumours from patients who lived beyond 3yrs (n = 16). Shrunken centroid classifiers were developed use the PAMR method [59], and cross-validation suggested the optimal classification was obtained using eight of the gene network hub RNAs (Figure S1A). This analysis allowed 85% (12/16) of patients alive after 3 yrs to be correctly classified, and 90% (9/10) of patients dead before 2 yrs to be correctly classified in a cross-validation experiment. Expression patterns for the eight RNAs relative to the patient classes are shown in Figure S1B.

In summary, Bayesian gene network analysis of the A375 microarray data identified hubs with children enriched for numerous biological functions. In metastatic melanomas, gene network hubs with downstream children enriched for cell cycle functions are strongly associated with patient prognosis. Hence these hubs are candidate biomarkers for cell cycle activity and patient prognosis. Additional candidates as prognostic markers were identified in a pilot class prediction experiment.

### Laboratory investigation of Bayesian gene network hubs

While many of the Bayesian gene network hubs are already well known in cancer biology, some hubs represented molecules that had not been well characterised in terms of their role in cancer cells. We subjectively selected three poorly characterised hubs for further study – all had sets of child RNAs that were significantly enriched (GATHER Bayes Factor ≥5 and p≤0.05) for functions that were easy to assess in the laboratory: (i) *ELMOD1* (231930_at) has only 8 gene network children, however all encode proteins associated with programmed cell death (GO:0012501 – *MARK4*, *NGFRAP1*, *PIK3R2*, *PRKCA*, *PRSS23*, *SEPT4*, *TIA1*, and *TUBB4*). (ii) *TMCO1* (210768_x_at) has 92 network children, a significant subset of which encode proteins associated with the GO annotation of apoptosis (GO:0006915 – e.g. *BIT1*, *CRADD*, *EBAG9*, *NOL3*, *PSEN2*, *SPATA4* and *SPIN2*), and (iii) UBE2S (202779_s_at) has 67 network children, a significant subset of which encode proteins associated with the cell cycle (GO:0007049 – e.g. *AURKB*, *BUB3*, *CCNF*, *CDK5RAP1*, *CHAF1B*, *CHEK2*, *GTSE1*, *KIF22*, *KIFC1*, *MAD2L1*, *RAD54L*, *RFC5*, *RNASEH2A*, *RPA1* and *RPA3*) and DNA damage response/repair (GO:0006974 – e.g. *CHAF1B*, *CHEK2*, *DDB2*, *GTSE1*, *KIF22*, *NEIL3*, *RAD54L*, *RFC5*, *RPA1* and *RPA3*).

**Figure 8:**
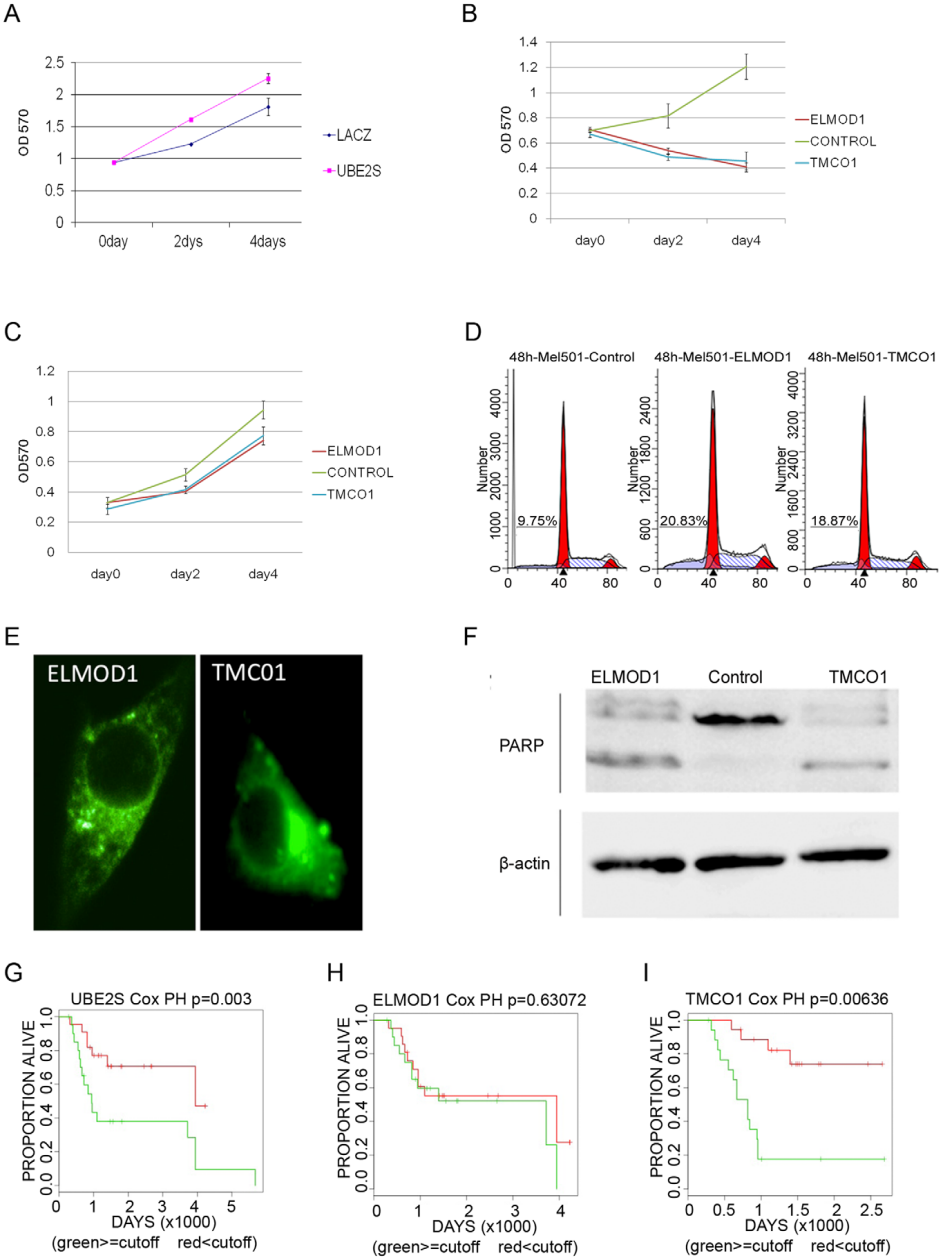
Laboratory investigation of gene network hubs. A–C, general cell biological effects of plasmid over-expression. Human 293T embryonic kidney cells (A and B) and human Mel501 melanoma cells (C) were transfected with *control* plasmids encoding lacZ and with plasmids encoding *UBE2S* (A), *ELMOD1* (B and C) and *TMCO1* (B and C). At 0, 2 and 4 days after the transfection, the number of viable cells was assessed using MTT assays. X-axes represent time in days while y-axis represent the OD570 absorbance (indicating viable cell number). Error bars represent standard deviation of the mean from four replicate wells. All graphs are representative of at least three independent experiments. D, Cell cycle analysis. 48 hours after transfection of plasmids into Mel501 cells, the cells were analysed by flow cytometry to identify the % cells in different phases of the cell cycle. Numbers show the percentage of hypodiploid cells. E, Fluorescent microscopy suggests that GFP-tagged over-expressed Elmod1 and TMC01 proteins have a punctate cytoplasmic distribution. F Western blotting indicates PARP cleavage in cells transfected by *Elmod1* and *TMC01* plasmids. 48 h after transfection of Mel501 melanoma cells with Elmod1 and TMC01 plasmids, protein lysates were analysed by Western blot using anti-β-actin and anti-PARP (a Caspase target degraded during apoptosis) antibodies. G–I, survival analysis in metastatic melanoma. Graphs compare survival of patients whose metastatic melanomas had above (green) or below (red) the 50^th^ percentile of the particular RNA expression in the Bogunovic 2009 data series [57]. All experimental data shown in panels A–F of this figure are representative of at least three independent experiments.

The coding regions of *ELMOD1*, *TMCO1* and *UBE2S* were amplified by proof-reading PCR from an A375 cell cDNA template and ligated into the *pcDNA3.3-TOPO TA* expression vector. After the sequence of these plasmids was checked, they were transfected into 293T epithelial cells and Mel501 melanoma cells, along with control plasmids encoding *lacZ*. Initial assessment using MTT assays suggested that transfection of 293T cells with the *UBE2S* expression plasmid caused a significant increase in cell numbers after 2 and 4 days, relative to *lacZ* control plasmids and untreated cells (Figure 8A, t-test p value ≤0.05). This is broadly consistent with the role predicted for *UBE2S* by the gene network as a positive regulator of many cell cycle children. In contrast, transfection with the *ELMOD1* and *TMCO1* expression plasmids caused a significant decrease in cell numbers relative to *lacZ* controls and untreated cells in 293T cells (Figure 8B, t-test p value ≤0.05), broadly consistent with the role predicted by the gene network as a positive regulator of many children associated with apoptosis. Transfection with the *ELMOD1* and *TMCO1* expression plasmids into Mel501 melanoma cells also caused a trend towards decrease in cell numbers relative to *lacZ* controls and untreated cells in 293T cells, however this was not statistically significant (Figure 8C, t-test p values  = 0.08 and 0.13 at 2 and 4 days, respectively).

In the course of our work we learned that *UBE2S* had already been characterised under another name [60]. This characterisation concurred with our own overexpression-MTS experiment, and showed that UBE2S increased the rate of the cell cycle by targeting and degrading the von Hippel-Lindau protein. Therefore, we did not follow *UBE2S* further in the laboratory. We studied the effect of transfection of *lacZ* control, *ELMOD1* and *TMCO1* expression plasmids on the cell cycle by flow cytometry of propidium iodine-stained cells. In both 293T cells (data not shown) and Mel501 cells (Figure 8D), transfection of the *ELMOD1* and *TMCO1* expression plasmids had no obvious effect on the proportion of cells with 2N and 4N DNA content, but it did substantially increase the proportion of cells with degraded DNA in the hypodiploid peak, consistent with the induction of apoptosis.

293T cells transfected with *lacZ* control, *ELMOD1* and *TMCO1* expression plasmids were then analysed by Western blotting with an antibody raised against the caspase-3 target PARP, which showed PARP cleavage in the cells transfected with the *ELMOD1* and *TMCO1* expression plasmids but not in the cells transfected with the control expression plasmid. Transfection of 293T cells with *GFP*-tagged *ELMOD1* and *TMCO1* expression plasmids using the *pIRESeGFPII* backbone indicated that the overexpressed proteins localise in structures within the cytoplasm (Figure 8E). The expression levels of *UBE2S* and *TMC01* but not *ELMOD1* RNA in metastatic melanomas appear to be significantly associated with the survival of patients (Figure 8G–I).

In summary, three relatively uncharacterised genes, which the gene networks predicted would influence the abundance of apoptosis-associated or cell cycle-associated RNAs, were found to alter apoptosis and cell number when overexpressed *in vitro*. The expression levels in metastatic melanoma tumours of two of these genes (*UBE2S* and *TMCO1*) appear to be significantly associated with the time to relapse in melanoma patients.

## Discussion

### Meta-analysis of melanoma microarray studies

RNA signatures from melanoma microarray studies have provided useful information about many aspects of the biology of this tumour type (see the papers summarised in Table 1). Therefore, at the start of this study we attempted a meta-analysis of published microarray data related to melanoma *progression*, *metastasis* and *prognosis*, hoping to identify consistent gene signatures for these clinical features. However, we found there was very little concordance between the different studies, despite the fact they appeared to have been carried out to a high standard. We noted that each of the published studies contained different patient groups, different tumour sites, and different histopathological tumour types. These differences may be in part responsible for the distinct gene signatures produced by the different microarray studies. A recently published review of melanoma microarray studies [61] has reached similar conclusions about discordance between melanoma microarray studies.

### siRNA-treated A375 cells appear to model some but not all transcriptional relationships present in melanoma tumours

Given the diversity of melanomas in patients discussed above, we proposed that rather than perform a meta-analysis of patient tumours, a more effective way to obtain new insights into melanoma biology could be to generate a microarray dataset in A375 cells, in which transcription factors and signalling molecules were targeted using siRNAs. We hoped that this approach would generate a dataset with controlled differences between siRNA-treated cultures, to increase our sensitivity for revealing meaningful molecular pathways. Similar approaches have been used successfully in other cancers to understand oncogenic signalling pathways. For example, Bild *et al*. transfected cultures of quiescent primary mammary epithelial cells with specific oncogenes and performed microarray analysis to identify clinically relevant oncogenic pathways in breast cancer [62], and the connectivity map project [63] also takes the approach of deeply studying cancer cell lines placed into in a large number of different “states” *in vitro*. The dataset produced by this experiment was analysed using whole genome Bayesian networks, and since this method is relatively new, in parallel using a simple hierarchical clustering method. Reassuringly, both methods identified similar co-expression clusters. It was interesting that eleven of the molecules previously implicated in melanoma pathogenesis (described in the introduction) were identified as hubs in the Bayesian gene networks generated from our A375 cell dataset, including: *BRAF*, *CCND1*, *RB1*, *PTEN*, *TYR*, *CDKN2A*, *and SOX10*. However, the interactions between these molecules that are known experimentally (at the level of either transcription or post-translational signalling) were in general not identified by the gene networks. It is possible that these interactions simply do not operate in cultured A375 cells, or that the 45 siRNA disruptions used in this study did not introduce sufficient variability in the expression of these molecules to allow latent relationships between them to be identified.

Like all *in vitro* cell work, our use of A375 cells, cultured in the laboratory potentially comes at the cost of losing biological validity. To assess the similarity between A375 cells and melanomas in patients at a transcriptional pathway level, we compared the RNA correlations within biologically-relevant gene sets identified across A375 cells with those identified across both primary and metastatic melanomas. We found that several gene sets (e.g. those related to the cell cycle) were approximately equivalently correlated across both the A375 cells and the clinical data. We identified other gene sets that were more frequently correlated in the clinical microarray data than in the A375 cell data, such as gene sets associated with immune response. Immune response plays a major role in melanoma biology [64] and has prognostic implications for melanoma patients [57], [65] and, as described in the introduction, therapies that modify immune pathways in melanoma hold great promise for a subset of melanoma patients. However, the fact that the transcriptional pathways associated with melanoma immune response and inflammation are not apparent in our A375 cell data limits our ability to study these biological processes using melanoma cell lines *in vitro*. This limitation is not surprising, given that the immune cells that participate in these pathways in tumours are absent from the A375 cell cultures. Other gene sets that were more frequently correlated in the clinical microarray data than in the A375 cell data include heparin sulphate proteoglycan synthesis and amino acid acetylation, as well as targets of the transcription factors MYC/MAX, POU3F2 and GCNF. It is possible that these processes/transcription factors are highly active in tumour stromal cells, and therefore are not identifiable in cultured A375 cells.

Hub genes are predicted by the Bayesian gene network to be regulators of the expression of their children. Experimental evaluation of the directional gene expression relationships between gene network parents and their children is beyond the scope of this study. Nevertheless, we followed up three hubs from the A375 cell gene networks that were not well characterised. These were chosen based on the enrichment of their downstream network children for functions (cell cycle and apoptosis) that could be easily examined in our laboratory and that were clinically relevant. It was encouraging that in the pilot experiments presented here, overexpression of RNAs encoding these hubs appeared to alter the specific functions associated with hub children. Further studies are now needed to confirm the levels of plasmid expression achieved and to define the mechanisms by which hub overexpression alters cell biology.

### Associations between the abundance of genes highlighted by the A375 gene networks and patient survival

We found that in metastatic tumours, the abundance of gene network hub RNAs enriched for children with cell cycle and DNA repair functions, but not several other functions, were frequently associated with patient survival. Some of the hub genes we identified had previously been associated with survival in the original Bogunovic *et al.* study of this dataset [57]. These findings fit well with the known role of cell proliferation pathways in melanoma progression. For example, mitotic incidence is the second most powerful prognostic factor after thickness for primary melanoma [66], and many of the inherited melanoma predisposition genes encode proteins involved in the cell cycle (see Introduction). The cell cycle clusters identified by the gene network, and the hubs that are predicted by the network to drive the expression of these clusters, may in the future assist selection of biomarkers for the prognosis of metastatic melanoma lesions, to supplement the assessment of histological grade as a prognostic indicator.

Our choice of the 45 siRNAs transfected into the A375 cells was in fact biased towards RNAs encoding proteins with specific functions. The GO database indicated that of the 45 siRNAs used, 21 were associated with regulation of the cell cycle (GO:0000074), 22 with regulation of transcription (GO:0045449), and 24 with regulation of metabolism (GO:0019222). Since gene network inference depends upon variation in gene expression between the siRNA-targeted cell cultures, large numbers of siRNAs related to proliferation may have increased the resolution of our gene networks for proliferation-associated transcriptional pathways, and therefore contributed to our identification of survival-associated cell cycle clusters. However, the effect of siRNA choice on gene network results may be complex, since we did not see dominant network clusters associated with the functions of metabolism and transcription, for which our siRNA set was also enriched. We are unsure why no strong relationship was seen between abundance of hub RNAs enriched for children with cell cycle functions and patient survival in the *primary* tumour data [58]. It is possible that the effect of cell cycle pathways on tumour biology is only significant in metastatic tumours. However, this seems unlikely since our supervised clustering analysis (Figure 5) showed similar correlations of cell cycle-associated gene sets in primary and metastatic melanomas. To clarify this issue, annotation of other previously published Affymetrix studies of primary melanomas with patient survival data would be useful.

Given the clear association between cell proliferation transcriptional modules identified in this study and patient prognosis, these modules have potential clinical usefulness. For example, an 8-gene classifier developed from Bayesian gene network hubs correctly classified the prognosis of 23/26 metastatic melanoma patients in a pilot cross-validation study (Figure S1). While this is very encouraging, given the very small number of patients that could be used for this class prediction, and the cross-validation strategy that therefore had to be employed, further studies using large independent test sets are now needed to validate this classifier. As another potential use, gene expression modules associated with specific drug targets may provide biomarkers to allow patient stratification. For example, the cell cycle-associated gene network hub *TYMS* is a target of 5-fluorouracil, a drug that has been studied in melanoma in the past [67] but has not proved generally successful in melanoma patient populations. It is possible that those melanoma patients with high expression of RNAs that are clustered with/downstream of *TYMS* in our analysis, indicating active molecular pathways involving *TYMS,* may be better candidates for topical 5-fluorouracil treatment than other patients.

### Assessment of the effects of cross-hybridisation

As described in the methods, we showed that, for one cluster of co-regulated RNAs in the A375 melanoma cell dataset, the highly correlated gene pairs were not conserved in a similar dataset generated in MCF-7 breast cancer cells (Figure S2A). This analysis was then repeated for all probe sets (Figure S2B and S2C). This implies that cell type-specific transcriptional pathways, and not cross-hybridisation, are the dominant driver of the RNA-to-RNA relationships on which this study was based. However, the extent of cross-hybridisation in Affymetrix data remains an area of debate, with some researchers suggesting that cross-hybridisation may cause problems for correlation-based analysis of microarray data [68], while others conclude that this is not the case, and that “the observed long-range correlations in microarray data are of a biological nature rather than a technological flaw” [69]. Our data supports this latter view, although further studies may be needed to address this issue fully. Cross-hybridisation will not be an issue when the techniques described here are applied to RNAseq data.

### Conclusion

In this study, we used siRNAs to knock down the abundance of 45 functionally important mRNAs in A375 melanoma cells. A variety of methods were then used to reverse engineer co-expression clusters and gene networks from this data. We identified several gene sets that were correlated both across siRNA-treated A375 cells *and* across melanomas from patients (e.g. gene sets associated with the cell cycle), as well as other gene sets that were correlated only across the clinical melanomas (e.g. gene sets associated with immune function). Several clusters enriched for cell cycle functions and the hubs upstream of these clusters in the gene networks were significantly associated with patient survival, suggesting new prognostic biomarkers, and underlining the importance of the transcriptional pathways that control the cell cycle for melanoma biology. Our analysis also illustrated the frequent co-expression of functionally-related RNAs. We hope that bioinformatic methods like those used here can work alongside traditional tumour biology studies to improve our understanding of melanoma and to derive new biomarkers and drug targets suited to the tumours of individual patients. In addition, we hope that the methods described here for estimating the correlation of genes that share the same biological functions will be useful to estimate the validity of cell culture models for specific aspects of other human diseases.

## Materials and Methods

### Cell culture and transfection

A375 melanoma cells [70] and HEK293T (293T) embryonic kidney epithelial cells were obtained from the American Type Culture Collection (ATCC, Manassas, USA). Mel501 cells [71] were provided by Dr Ruth Halaban (Yale University School of Medicine, New Haven, CT). A375 cells were chosen for the *in vitro* functional genomics experiments described here since their transcriptome appears to be moderately representative of clinical melanoma tumours; for example they are positioned close to several tumours in a multidimensional scaling analysis [31]. A375 and 293T cells were cultured in Dulbecco's Modified Eagle Medium (DMEM) supplemented with 10% foetal calf serum. Mel501 cells were cultured in opti-MEM medium (Invitrogen, Carlsbad, USA) supplemented with 7% foetal calf serum. All cells were maintained at 37°C in a fully humidified atmosphere containing 5% CO2. For transfection with siRNAs (20 nM final concentration, Dharmacon siGenome Smartpools, Dharmacon, Lafayette, USA) cells were seeded in 6-well plates, and the following day when cell density had reached 30% confluence, cells were washed and media replaced with 1 mL of opti-MEM containing 30 µl Oligofectamine (Invitrogen, Carlsbad, USA) and 10 nM siRNA duplexes. 48 hours after transfection cells were harvested using TRIzol (Invitrogen, Carlsbad, USA) and RNA extracted using the RNeasy RNA Extraction system according to the manufacturer's instructions (Qiagen, Hilden, Germany). The 45 siRNA transfections into A375 cells were performed in three experimental batches. RNA quality was confirmed using an Agilent 2100 bioanalyser. For transfection with plasmids, the same procedure was followed except that 5 ul Lipofectamine 2000 reagent (Invitrogen, Carlsbad, USA) and 2 ug plasmid were mixed in 1 mL opti-MEM medium and incubated at room temperature for 15 min before being added to the washed cells for 48 hours.

### Microarray analysis and data processing

Biotin-labelled cRNA was generated and hybridised to Affymetrix Human Genome U133plus 2.0 microarrays following the manufacturer's instructions (Affymetrix, Santa Clara, USA). All microarray data used in this manuscript is MIAME compliant and has been deposited in GEO. The GEO accession number for microarray data from siRNA-treated A375 cells is GSE31534. The GEO accession number for microarray data from siRNA-treated MCF-7 cells is GSE 31912. Array analysis was performed using the statistical framework ‘R’ (http://cran.r-project.org/). All microarrays passed quality control using the ‘AffyQCreport’ R package. Microarray ‘CEL’ files from the siRNA-treated A375 cells, and from previously published studies, were normalised using the Robust Multichip Averaging (RMA) algorithm [72] provided by the R ‘affy’ package. To remove any possible batch effects we scaled each probe set based on its median expression in each experimental batch. Gene lists were tested for enrichment of particular functional categories using the GATHER web tool [49], as well as using the Ingenuity Pathways Analysis (IPA) software (http://www.ingenuity.com/).

Spearman rank correlation coefficients, which are based on the correlations between the ranks of variables, were generated using the ‘cor’ function in the R base package. Whole-genome Bayesian gene networks were reverse engineered from the siRNA-treated A375 cell microarray data by estimating large numbers of sub-networks in parallel that were later amalgamated, as described [47], using massively parallel supercomputers at the Tokyo University Human Genome Center. Kaplan-Meier survival analysis with both log-rank significance tests and significance tests using Cox proportional hazards models were performed using the R ‘survival’ package (http://cran.r-project.org/web/packages/survival/). Class prediction was performed using shrunken centroid classifiers with the “pamr” R package [59].

### Meta-analysis of published melanoma microarray datasets

For all “cel” files quality control was performed using the ‘AffyQCReport’ package [73] in R. Re-normalisation of all arrays together was in all cases performed from raw data in.cel files using the RMA method with background correction, and differential expression was analysed using LIMMA (Linear Models for Microarray Data) [74]. The “metaMA” package in R [75] was used to perform statistical meta-analysis. The GATHER web tool [49] was used to identify whether any lists of differentially expressed RNAs were enriched for the targets of specific transcription factors (using the TRANSFAC Pro database v8.0, [50]), or for molecules with a common function (the Gene Ontology (GO) database, [51]). The PCOT2 multivariate analysis method in R [42] was also used to identify correlated differential expression KEGG gene sets [76]. When composite data sets were generated from multiple published melanoma microarray studies, to remove study cohort effects we scaled each probe set based on its median expression in each study.

### Hierarchical clustering

All 54,000 probe sets on the A375 cell microarrays were hierarchically clustered using Ward's method [77] with dissimilarities between observations calculated using 1−|ρ| to allow positively and negatively correlated genes to be included in the same cluster. Clusters were chosen so that: cluster size was >5 probe sets, the minimum correlation between any two probe sets within a cluster was ≥0.4, and the median correlation of all possible combinations of the members of each cluster was ≥0.5. The 200 clusters selected contained in total 1,753 probe sets; 35% of the clusters contained 5–10 probe sets, 9% contained 10–20 probe sets, with 13 clusters containing ≥30 probe sets. For over-representation analysis using the GATHER web tool, significant enrichment of cluster members was said to have occurred when: Bayes Factor≥5 and permutation p≤0.05. For this descriptive investigation of the A375 cell data, the cutting of the dendrogram into 200 clusters and the parameters used for cluster membership filtering were arbitrary choices. However, trials using different cluster numbers and parameters for cluster filtering did not produce clusters that were significantly enriched for any additional gene sets. When we repeated this clustering using only those probe sets with median expression of ≥1.5x the 3′ BioB probe set (a probe set that can be used as an indicator of the noise threshold), five clusters associated with the cell cycle and seven associated with transcription factor targets were identified but no additional GO paths or transcription factor motifs for which clusters were enriched were identified.

### Evaluation of cross-hybridisation artefacts

It has been suggested that a fraction of the probe sets in Affymetrix microarrays may cross-hybridise with multiple mRNA transcripts, which could lead to spurious clustering and gene network relationships. Therefore, we calculated the Spearman's correlation coefficients between all possible combinations of probe sets from the cluster shown in Figure 3 across: (i) our A375 siRNA Affymetrix Human Genome U133 plus 2.0 dataset and (ii) an unpublished Affymetrix Human Genome U133 plus 2.0 dataset from our laboratory, in which we have used a set of 70 siRNAs to target MCF-7 breast cancer cells. Given that the identical microarray platform was used in the A375 and MCF-7 siRNA datasets, if cross-hybridisation was the dominant driver of the clustering observed in the A375 cells, then we would expect to see similarly high correlations between the same probe sets in the MCF-7 cells. In fact, we found that the high correlations observed between probe sets in the A375 cells were largely absent from the MCF-7 data (Figure S2A). We then repeated this on a whole-genome scale, by calculating the Spearman's ρ in the MCF-7 data for the 54,681 probe set pairs that had Spearman's |ρ|≥0.8 in the A375 data (Figure S2B), and by calculating the Spearman's ρ in the A375 data for the 184,911 probe set pairs that had Spearman's |ρ|≥0.8 in the MCF-7 data (Figure S2C). These analyses suggested that Affymetrix Human Genome U133 plus 2.0 microarray probe set pairs that were highly correlated in data from A375 cells were in general not highly correlated in data using the same microarray platform from MCF-7 cells and *vice versa*, suggesting that cross-hybridisation between Affymetrix probe sets was not the most significant driver of the clustering we observed across the A375 dataset.

### Whole genome Bayesian gene network analysis

This was carried out using massively parallel supercomputers at Human Genome Center of the University of Tokyo as previously described [45]. In brief, this method uses a heuristic algorithm called *the neighbor node sampling & repeat (NNSR)* method to estimate 100,000 overlapping small sub-networks selected from the intermediate global network structure, which is improved by the estimated sub-networks during the method. Edges that were present in at least 20% of these sub-networks were combined into a final 1,645,882 edge gene network for analysis (can be downloaded, with a brief explanatory file, from http://www.bioinformatics.auckland.ac.nz/doc/project_data/Supplementary_FIle_1.txt).

**Table 4:** Primers.

Primer orientation	RNA target	Sequence (5′ to 3′)
forward	ELMOD1	CACCATGAAGCACTTCCTGAGAATG
reverse	ELMOD1	GGATCCCTACATGTTGATTAAACCTTCCG
forward	UBE2S	CACCATGAACTCCAACGTGGAGAAC
reverse	UBE2S	TCACGGTGGAAGGAGGAA
forward	TMCO1	CACCATGAGCACTATGTTCGCGG
reverse	TMCO1	TCAAGAGAACTTCCCAGAAGGA

To illustrate the information underlying this network, Figure S3 explores the relationships between those gene network parents that were targeted by siRNA when generating the data set from which the gene networks were inferred, and their 1,800 gene network children. Although this analysis was by necessity performed on the same data set from which networks were inferred, the Bayesian gene network inference method used does not utilise information about the effects of the siRNA treatments on individual probe sets. Correlations between these parents and their children are significantly larger then correlations between randomly chosen nodes (Figure S3A). These children show a trend to be down-regulated by parent knockdown when parent and child correlate positively across the dataset, and to be up-regulated by parent knockdown when parent and child correlate negatively (Figure S3B–D). The regulation of child abundance after parent knockdown was generally small in magnitude, consistent with the expected dilution of the effect of knocking down any single parent by the undiminished effects of the remaining parents that were not knocked down. This Bayesian network method was primarily used in this study to identify co-expressed clusters rather that directional regulation. Future experimental evaluation of directional network predictions will be interesting but this is beyond the scope of this study.

### Cloning

Coding regions were amplified using Platinum Taq DNA Polymerase (Invitrogen, Carlsbad, USA) from cDNA that had been reverse transcribed from A375 cell RNA, then cloned directly into the pcDNA3.3-TOPO-TA expression vector. Ligation products were used to transform One-shot Top10 competent cells (Invitrogen, Carlsbad, USA) and colonies selected by PCR using gene-specific primers. Plasmid DNA was extracted using a Zyppy Plasmid Miniprep Kit (Zymo, Orange, USA) and the inserts sequenced on both strands. Plasmids for transfection were prepared using a QIAGEN endo-free Plasmid Maxi Kit according to the manufacturer's instructions (Qiagen, Hilden, Germany). The oligonucleotide primers used for cloning are described in Table 4.

### Analysis of culture growth, the cell cycle and apoptosis

MTT (3-(4,5-Dimethylthiazol-2-yl)-2,5-diphenyltetrazolium bromide) colorimetric assays were used to estimate viable cell number according to the manufacturers instructions (Invitrogen, Carlsbad, USA). Briefly, A375, Mel501 and 293T cells were seeded in 96-well plates at 3,000, 6,000 and 12,000 cells/well respectively. 10 µl of MTT (5 mg/ml) was added into each well; after 4 h incubation at 37°C 100µl of lysis buffer was added and plates were incubated at 37°C overnight. The next day the plates were read at OD570. Each condition was analysed in six replicate wells and all experiments were repeated at least three times.

For cell cycle analysis, cells in each well of a 6-well plate were trypsinised, washed three times by ice cold PBS then fixed in 3 ml of 70% ethanol in PBS added dropwise while vortexing. Cells were stained with 50 µg/ml propidium iodine (PI), 0.1 mg/ml RNase A, 0.05% (v/v) Triton X-100 at 37°C for 40 min then assayed by Flow Cytometry, with the results analysed using ModFit LT software (Verity, Topsham, USA) according to the manufacturer's instructions. All cell cycle analysis experiments were repeated at least three times.

For Western blotting to identify caspase activation during apoptosis, cells were trypsinised and washed three times with ice cold PBS, then pellets were mixed with lysis buffer (50 mM Tris.NaCl, 150 mM Na.Cl, 1% (v/v) Triton X-100, 1 mM EDTA, 1 mM EGTA and 1% (v/v) Protease Inhibitor Cocktail (“Complete”, Roche, Basel, Switzerland)) and incubated for 30 min on ice. Lysates were then micro-centrifuged at 4°C at 13,000 rpm for 15 min then 20 µg loaded into each well of 10% SDS-PAGE gels, separated, then transferred to BioTrace NT nitrocellulose membranes (PALL, Port Washington, USA). Membranes were blocked for 2 h at room temperature in blocking buffer (Tris-buffered saline containing 1% (v/v) Tween-20 and 5% (w/v) skim milk powder) and then incubated with anti-Poly ADP-ribose polymerase (PARP) antibody (Cell Signalling Technology, Danvers, USA) at 4°C overnight. The next day the membranes were washed in blocking buffer and incubated with goat anti-rabbit IgG–peroxidase antibody, washed three times in blocking buffer, then incubated with ECL plus reagents (GE healthcare, Pittsburgh, USA) for 5 min before scanning. All PARP Western blotting experiments were repeated at least three times.

## Supporting Information

Figure S1 **Class prediction based on the gene network hub probe sets.** (A) Shrunken centroid classifiers were developed and assessed by cross-validation using eight gene network hub probe sets. (B) For these eight probe sets, the normalised expression signals in metastatic melanoma tumours from the Bogunovic et al. (2009 [57]) dataset (y-axis) are plotted across the 26 tumours (x-axis). Green represents patients who died before 2 yrs and red patients who lived beyond 3 yrs.
(TIF)

Figure S2 **Affymetrix probe set-to-probe set correlations are not conserved between cell types.** (A) The kernel density plot shows the distribution of Spearman's correlation coefficients between all members of an E2F1-associated A375 cell-derived cluster (which was shown in Figure 3) in the A375 cell data (red) and in a similar MCF-7 cell dataset (green). (B) Spearman's correlation coefficients were calculated in the MCF-7 data (green) for the 54,681 probe set pairs that had |Spearman's correlation coefficients| ≥0.8 in the A375 data (red). Spearman's correlation coefficients were calculated in the A375 data (red) for the 184,911 probe set pairs that had |Spearman's correlation coefficients| ≥0.8 in the MCF-7 data (green).
(TIF)

Figure S3 **Relationships between gene network parents that were targeted by siRNA and their gene network children.** (A) The distribution of Spearman's correlation coefficients between parents that were targeted by siRNAs and their 1,800 gene network children is shown in red. The distribution of Spearman's correlation coefficients between ten randomly chosen sets of 1,800 genes is shown in grey as a control. (B) For each of the 1,800 gene network children shown in A, the ratio of (expression after siRNA knockdown of the parent) to (median expression across all microarrays) was calculated. This ratio will always be < = −1 or > = 1). For all 1,800 parent-child edges, this ratio (y-axis) was plotted against Spearman's correlation (x-axis). This shows a trend for the gene network children of parents targeted by siRNAs to be down-regulated after parent knockdown when parent and child correlate positively, and to be up-regulated after parent knockdown when parent and child correlate negatively. C and D show the distributions of fold change after parent knockdown for those network edges where parent and child were positively and negatively correlated, respectively.
(TIF)

Table S1 **The hubs in the Bayesian network with children significantly enriched (Bayes Factor ≥5 and p≤0.05) for functions related to cell cycle, mitosis or proliferation.** Hub probe ID and hub official gene symbol (OGS) are given in the first two columns. The GATHER Bayes Factor and the probability of obtaining this Bayes factor due to chance are shown in columns 4 and 5. Column 6 lists the children of the gene network hub that have the enriched annotation listed in column 3. Column 7 shows the Cox proportional hazards (coxPH) survival p-value for the association of hub RNA abundance in metastatic tumours with patient survival, with those hubs with p-values ≤0.05 highlighted in yellow.
(XLSX)

Table S2 **A375 Bayesian network hubs that have children significantly enriched for cell cycle functions.** Affymetrix probe set ID (parent probe ID) and official gene symbol (parent OGS) are shown in columns 1 and 2. The number of children of these hubs in theA375 Bayesian gene network is shown in column 3. Of these children, the number and % that have |Spearman's ρ| ≥0.4 with their parents are shown in columns 4 and 5, respectively.
(XLSX)

Table S3 **Conserved correlations between the hub DTL (probe ID 222680_s_at) and its children between the A375 in vitro siRNA dataset and metastatic tumours in patients of the Bogunovic study.** The gene network parent (222680_s_at) and gene network child probe set ID and OGS are given in the first 4 column. Column 5 shows the |Spearman's ρ| between the parent and child across the A375 siRNA dataset, while column 6 shows the |Spearman's ρ| between the parent and child across the Bogunovic metastatic tumour dataset. In columns 5 and 6 |Spearman's ρ| ≥0.4 are highlighted in orange. Columns 7 and 8 show the Cox proportional hazards p-value for association between probe set abundance in metastatic tumours and patient survival for parent and child, respectively. In columns 7 and 8 p≤0.05 are highlighted in green. (Note that the presence of some gene network edges with relatively low |correlation coefficients|, as seen here, is expected in Bayesian gene networks due to some network edges having high partial residual correlations even though they have low correlations across the data.
(XLSX)
