# Supplementary figures and images for: Cell Cycle Gene Networks Are Associated with Melanoma Prognosis
Source: PLoS One. 2012 Apr 20;7(4):e34247. doi: 10.1371/journal.pone.0034247 (PMC3335030; doi:10.1371/journal.pone.0034247)

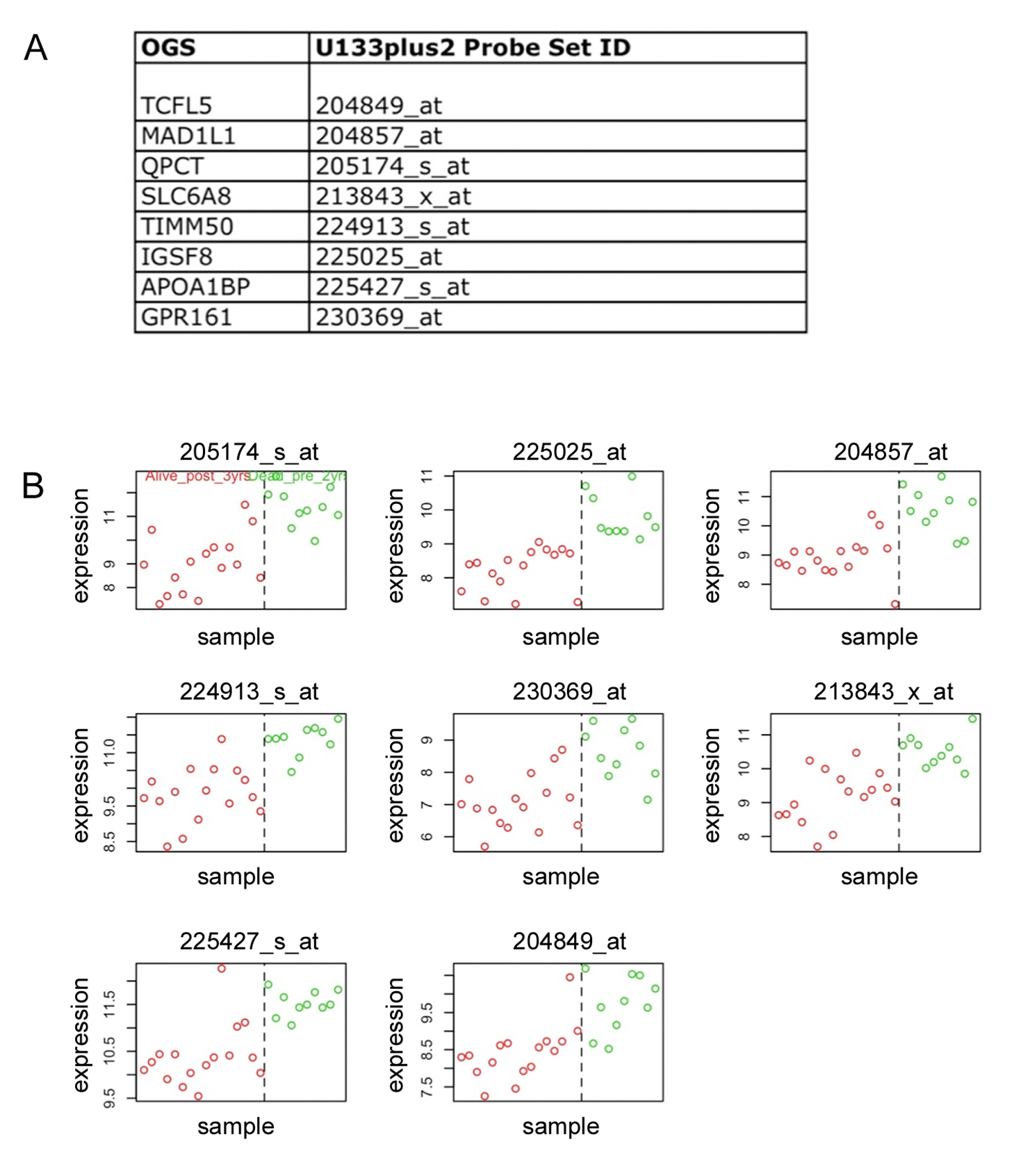

Supplement: Figure S1 — Class prediction based on the gene network hub probe sets. (A) Shrunken centroid classifiers were developed and assessed by cross-validation using eight gene network hub probe sets. (B) For these eight probe sets, the normalised expression signals in metastatic melanoma tumours from the Bogunovic et al. (2009 [57]) dataset (y-axis) are plotted across the 26 tumours (x-axis). Green represents patients who died before 2 yrs and red patients who lived beyond 3 yrs. (TIF) [file pone.0034247.s001.tif]

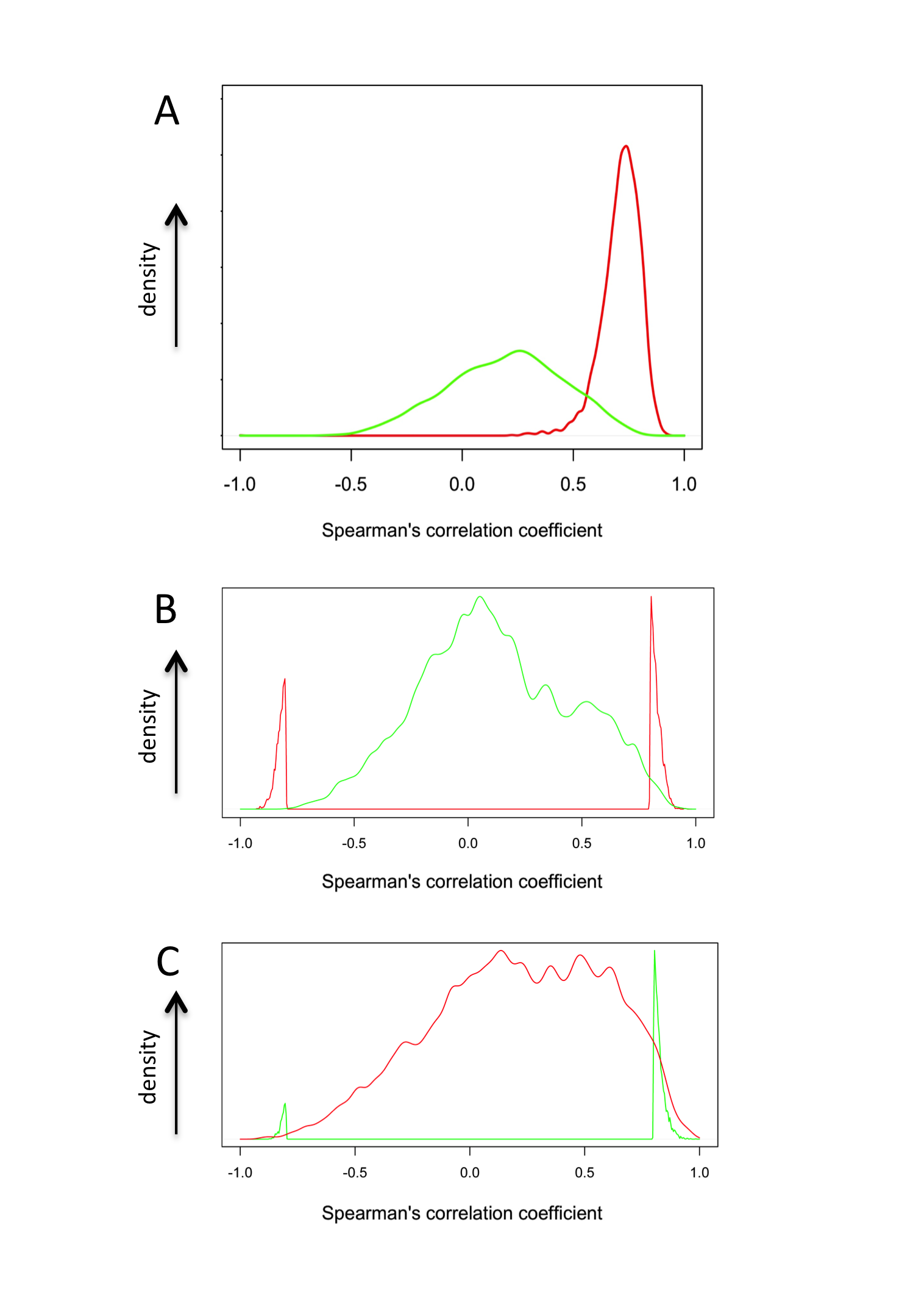

Supplement: Figure S2 — Affymetrix probe set-to-probe set correlations are not conserved between cell types. (A) The kernel density plot shows the distribution of Spearman's correlation coefficients between all members of an E2F1-associated A375 cell-derived cluster (which was shown in Figure 3) in the A375 cell data (red) and in a similar MCF-7 cell dataset (green). (B) Spearman's correlation coefficients were calculated in the MCF-7 data (green) for the 54,681 probe set pairs that had |Spearman's correlation coefficients| ≥0.8 in the A375 data (red). Spearman's correlation coefficients were calculated in the A375 data (red) for the 184,911 probe set pairs that had |Spearman's correlation coefficients| ≥0.8 in the MCF-7 data (green). (TIF) [file pone.0034247.s002.tif]

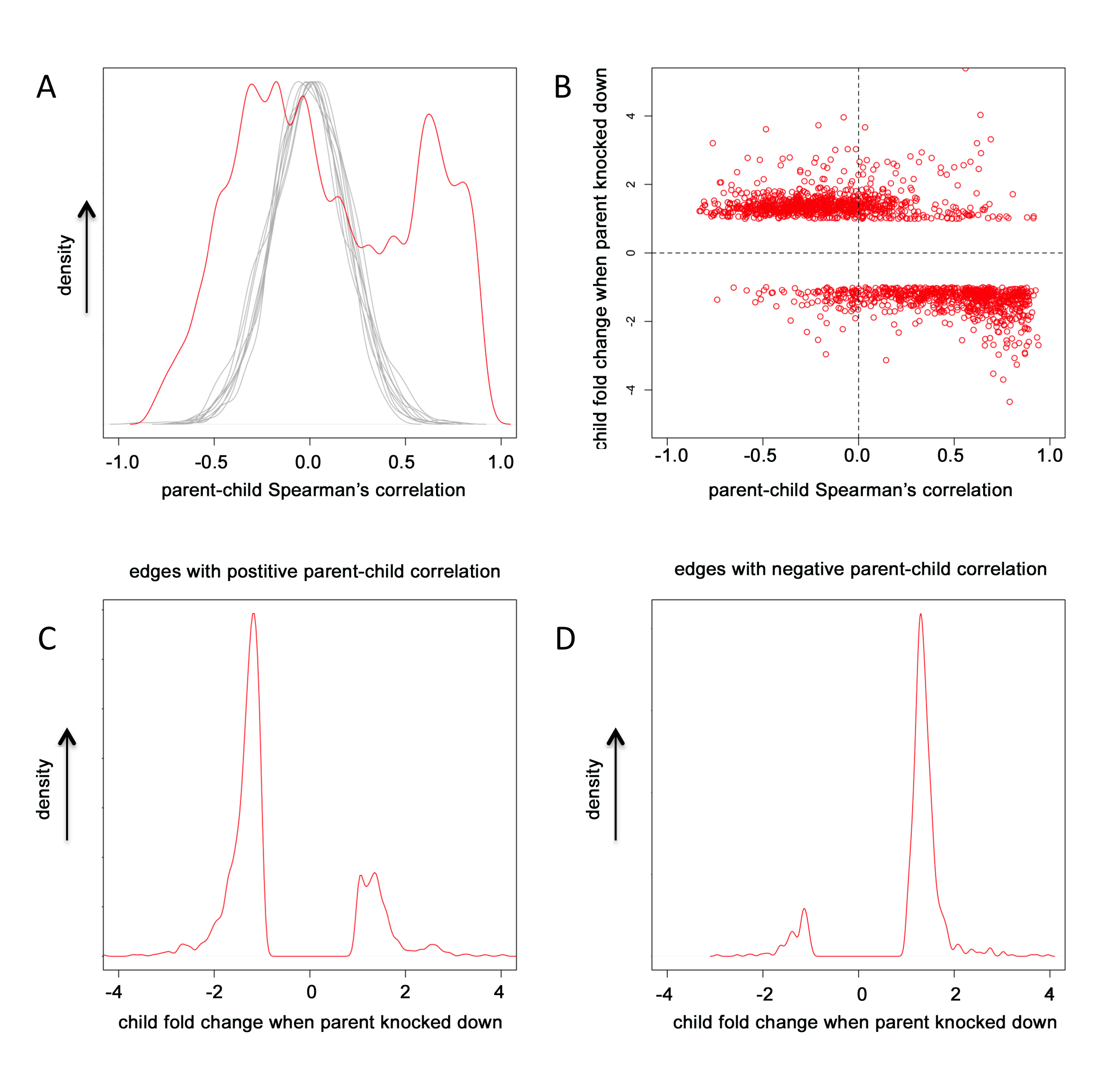

Supplement: Figure S3 — Relationships between gene network parents that were targeted by siRNA and their gene network children. (A) The distribution of Spearman's correlation coefficients between parents that were targeted by siRNAs and their 1,800 gene network children is shown in red. The distribution of Spearman's correlation coefficients between ten randomly chosen sets of 1,800 genes is shown in grey as a control. (B) For each of the 1,800 gene network children shown in A, the ratio of (expression after siRNA knockdown of the parent) to (median expression across all microarrays) was calculated. This ratio will always be < = −1 or > = 1). For all 1,800 parent-child edges, this ratio (y-axis) was plotted against Spearman's correlation (x-axis). This shows a trend for the gene network children of parents targeted by siRNAs to be down-regulated after parent knockdown when parent and child correlate positively, and to be up-regulated after parent knockdown when parent and child correlate negatively. C and D show the distributions of fold change after parent knockdown for those network edges where parent and child were positively and negatively correlated, respectively. (TIF) [file pone.0034247.s003.tif]
